# Clinical and Radiological Features of a Large Intracranial Hydatid Cyst: A New Case Report

**DOI:** 10.1155/crdi/4791747

**Published:** 2026-07-29

**Authors:** Kazem ghaemi, Mahmoodreza behravan, Soudabe eshaghi, Omid shafaee, Amir Tavakoli kareshk

**Affiliations:** ^1^ Department of Neurosurgery, Birjand University Faculty of Medicine, Birjand, Iran; ^2^ Infectious Diseases Research Center, Birjand University of Medical Sciences, Birjand, Iran, bums.ac.ir

**Keywords:** Birjand, brain hydatid cyst, case report, child, *Echinococcus granulosus*

## Abstract

Intracranial hydatid cyst is a rare but potentially fatal parasitic infection caused by the larval stage of *Echinococcus granulosus*. This report details a case of an intracranial hydatid cyst in an 11‐year‐old boy who presented with progressive headaches, nausea, vomiting, urinary incontinence, and tachycardia. Physical examination and initial laboratory studies were unremarkable, with no eosinophilia noted. Neuroimaging revealed a large cystic lesion in the left temporal lobe with significant mass effect. While initial differential diagnoses included various cystic lesions, the imaging characteristics were most consistent with a hydatid cyst. Surgical management involved a standard craniotomy and the Dowling technique for cyst removal, emphasizing meticulous technique to avoid rupture. The patient recovered well postoperatively with no neurological deficits and was discharged on albendazole therapy. This case underscores the importance of considering hydatid cyst in the differential diagnosis of intracranial cystic lesions, particularly in endemic areas with relevant exposure history.

## 1. Introduction

Hydatid disease, caused by the larval stage of the tapeworm *Echinococcus granulosus*, remains a significant public health concern in many endemic regions worldwide, including Iran. The infection occurs when humans accidently ingest parasite eggs excreted in dog feces, leading to cyst formation primarily in the liver and lungs [1]. Iran’s extensive rural and pastoral communities, where close contact between humans, dogs, and livestock is common, have long been recognized as hyperendemic areas for this zoonotic infection [2]. While the liver (70%–75%) and lungs (15%–20%) are the most common sites of hydatid cyst development, approximately 5%–10% of cases involve other organs through systemic dissemination [3, 4]. Cerebral hydatidosis represents an uncommon but clinically important manifestation, accounting for only 1%–2% of all reported cases. Interestingly, intracranial involvement shows a distinct epidemiological pattern, with children and young adults being disproportionately affected compared to adults, likely due to the greater compliance of the pediatric skull and brain parenchyma allowing gradual cyst expansion [5]. Epidemiological studies indicate that the mean age of patients at diagnosis is typically in the third to fourth decades of life, although children and adolescents represent a considerable proportion of cases in highly endemic regions. Iran continues to report a substantial number of surgical and clinically managed CE cases annually, reflecting an ongoing transmission cycle between dogs and livestock [6]. Cerebral hydatid cysts are typically primary, solitary, and supratentorial, with the parietal and temporal lobes being the most frequent locations. These lesions expand slowly and progressively, often remaining asymptomatic until they reach sufficient size to cause mass effect or increased intracranial pressure. When symptomatic, patients commonly present with persistent headaches, nausea, vomiting, focal neurological deficits, and seizures [7]. Seizures represent a particularly important presenting symptom when cysts involve cortical regions such as the temporal lobe, where they irritate adjacent epileptogenic brain tissue. Neuroimaging plays an indispensable role in diagnosis. Computed tomography (CT) typically reveals well‐defined, spherical, nonenhancing cystic lesions with contents isodense to cerebrospinal fluid. Magnetic resonance imaging (MRI) provides superior characterization, demonstrating low signal intensity on T1‐weighted images and high signal intensity on T2‐weighted images, typically without perilesional edema or contrast enhancement. These characteristic findings help distinguish hydatid cysts from brain abscesses, cystic tumors, and other intracranial lesions [8].

Surgical resection remains the definitive treatment, with complete cyst removal without intraoperative rupture being the goal to prevent anaphylactic reactions and secondary dissemination. In this report, we present a case of a large (6.5 cm) intracranial hydatid cyst in the left temporal lobe of an 11‐year‐old boy from Siujan village, Khusf County, South Khorasan, Iran, who presented with a transient loss of consciousness consistent with increased intracranial pressure. We highlight the importance of considering this diagnosis in pediatric patients from endemic regions and report on the patient’s successful recovery over a 1‐month postoperative follow‐up period.

## 2. Case Presentation

### 2.1. Patient Information

This case involves an 11‐year‐old boy residing in a rural area (Siujan village, Khusf County, South Khorasan, Iran). His exposure history included frequent contact with domestic dogs and consumption of unwashed fallen jujube fruits and vegetables from the soil. Notably, his grandmother had a history of extensive hydatidosis, although further details were pending. Considering the large size of the intracranial cyst and the young age of the patient, it is likely that the infection was acquired several years prior to diagnosis. Previous studies suggest that hydatid cysts generally grow slowly, with an estimated growth rate of approximately 1–5 cm per year, although this rate may vary depending on host and parasite factors. Therefore, the size of the cyst in this patient may indicate that the infection was acquired during early childhood.

### 2.2. Clinical Presentation

The patient presented with a ∼2‐month history of progressively worsening episodic headaches. These headaches were associated with nausea, repeated vomiting, and episodes of tachycardia. During severe headache attacks, he experienced urinary incontinence. Importantly, there was no witnessed tonic–clonic activity, postictal confusion, or other features suggestive of an epileptic seizure. The night before admission, he experienced a transient loss of consciousness resembling a syncope‐like episode rather than a seizure. Overall, the clinical presentation was more consistent with symptoms related to increased intracranial pressure than with new‐onset seizures. Initial treatment for presumed migraine proved ineffective.

### 2.3. Physical Examination

On physical examination, the patient’s Glasgow Coma Scale (GCS) score was 15, indicating full consciousness. His pupils were normal and reactive to light. Importantly, no focal neurological deficits were reported, suggesting that the mass effect had not yet caused focal neurological impairment.

### 2.4. Laboratory and Neuroimaging Findings

**FIGURE 1:**
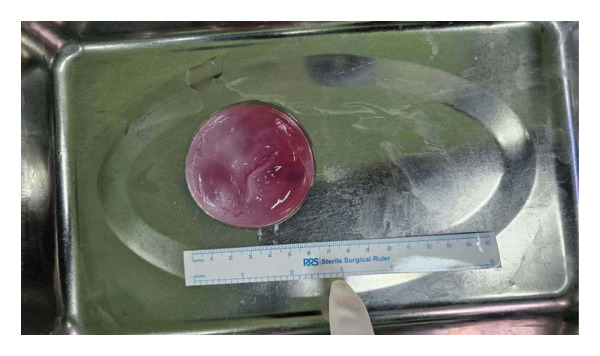
Surgical excision of the brain hydatid cyst.

**FIGURE 2:**
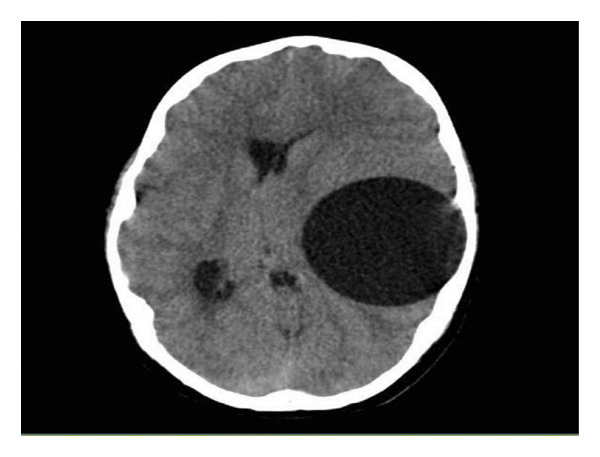
Brain MRI shows a large brain hydatid cyst on the left temporal lobe.

Routine laboratory investigations, including complete blood count (CBC), urinalysis (U/A), and biochemical tests, were all within normal limits. Specifically, there was no eosinophilia, which can sometimes be present in parasitic infections. Neuroimaging via CT and MRI of the brain identified a large unilocular, well‐defined cystic lesion in the left temporal lobe, measuring approximately 6.5 cm in maximal diameter (Figure 1). This lesion caused a midline shift of 2.5–3 mm and exhibited minimal perilesional edema. MRI signal characteristics showed the cyst to be T1 hypointense, T2 hyperintense, FLAIR hypointense, and DWI hypointense, findings compatible with the high water content typical of a hydatid cyst (Figure 2). Although the World Health Organization Informal Working Group on Echinococcosis (WHO‐IWGE) classification is primarily established for hepatic hydatid cysts, the imaging features of this cerebral lesion, specifically a well‐defined, unilocular, and homogeneous cystic appearance, are structurally analogous to the active CE1 stage. The absence of detached internal membranes or a water‐lily sign distinguishes this lesion from the Transitional stage CE3a. Molecular characterization of *E. granulosus* was performed to confirm the species. DNA was extracted from the cyst material obtained during surgery, and molecular analysis was conducted using the polymerase chain reaction (PCR) amplification of specific mitochondrial gene targets. The results confirmed the isolate as *E. granulosus*.

### 2.5. Radiologic Differential Diagnosis and Final Diagnosis

Initially, the radiologic differential diagnoses included arachnoid cyst, epidermoid cyst, dermoid cyst, and meningioma with cystic degeneration. However, the specific findings on CT/MRI, such as sharp margins, FLAIR suppression, DWI hypointensity, the absence of solid components or fat, and minimal edema, strongly favored an intracranial hydatid cyst, leading to the exclusion of other differentials. Systematic extracranial screening was limited; however, no clinical or available imaging evidence suggested hepatic or pulmonary involvement at the time of evaluation.

### 2.6. Molecular Confirmation

For molecular confirmation of the diagnosis, genomic DNA was extracted directly from the aspirated cyst fluid. PCR amplification was subsequently performed targeting the Mitochondrial cytochrome c oxidase subunit 1 (COX‐1) gene. The successful amplification of the target gene confirmed the diagnosis of *E. granulosus* at the molecular level.

### 2.7. Surgical Management and Postoperative Course

Surgical management was undertaken via a standard craniotomy over the left temporal region using the Dowling technique. Controlled cerebrospinal fluid (CSF) drainage was performed to reduce intracranial pressure. Crucially, direct manipulation of the cyst was avoided, and gravity‐assisted delivery was employed without any traction to prevent inadvertent rupture. The operative time was 6 h, and no intraoperative complications occurred. The cyst was successfully removed intact and unruptured. Following surgery, the patient experienced no postoperative neurological deficits. A postoperative CT scan confirmed a normal study with no residual lesion, hemorrhage, or hydrocephalus. The patient’s body weight was approximately 35 kg. The patient was hospitalized for 11 days, and postoperative albendazole therapy was initiated at a dose of 200 mg twice daily (approximately 10–15 mg/kg/day, consistent with recommended pediatric dosing). Antiparasitic treatment was continued after discharge, with a planned duration of 3 months to reduce the risk of recurrence. Liver function tests and CBCs were scheduled to be monitored periodically during treatment to detect potential drug‐related toxicity. A follow‐up imaging plan was established, including periodic neuroimaging, to assess for recurrence or residual disease. During postoperative follow‐up, the patient remained clinically stable with no evidence of recurrence, residual lesion, seizure activity, neurological deficit, or treatment‐related adverse events throughout the 1‐month follow‐up period. At the time of discharge, his condition was stable, and he was symptom‐free.

### 2.8. Clinical Timeline (CARE Framework)

∼2 months before admission: The patient developed progressively worsening episodic headaches associated with nausea, vomiting, and occasional tachycardia.

1 day before admission: The patient experienced a transient loss of consciousness resembling a syncope‐like episode during a severe headache attack.

Day of admission: The patient was admitted for further evaluation due to worsening symptoms and failure of initial treatment for presumed migraine.

Hospital evaluation: Brain imaging revealed a cystic lesion in the left temporal region consistent with a hydatid cyst.

Surgery: A left temporal craniotomy was performed using the Dowling technique, and the cyst was removed intact without rupture.

Postoperative period: Postoperative CT scan showed no residual lesion, hemorrhage, or hydrocephalus. Albendazole therapy was initiated.

Discharge: The patient was discharged in stable condition without neurological deficits.

Follow‐up: During the 1‐month postoperative follow‐up, the patient remained clinically stable with no evidence of recurrence, seizures, or treatment‐related adverse effects.

## 3. Discussion

Intracranial hydatid cysts, caused by the larval stage of *E. granulosus*, represent a rare but important cause of intracranial space–occupying lesions, particularly in children living in endemic regions. This case emphasizes the importance of considering hydatid cysts in the differential diagnosis of pediatric intracranial cystic lesions when patients present with progressive symptoms of increased intracranial pressure in an endemic setting. The patient’s progressive headache, nausea, and vomiting, together with his exposure to domestic dogs and consumption of unwashed fruits and vegetables, are consistent with the typical epidemiological risk factors associated with echinococcosis [9]. The positive family history of hydatidosis further increased the clinical suspicion. Although hydatid disease most commonly involves the liver and lungs, neurological manifestations may occur when larvae reach the central nervous system [10]. Neuroimaging plays a central role in the preoperative diagnosis and differential diagnosis of intracranial hydatid cysts. In this case, MRI demonstrated a well‐defined cystic lesion with T1 hypointensity and T2 hyperintensity, thin smooth walls, and absence of internal septations or solid components, features strongly suggestive of a hydatid cyst. These imaging findings help differentiate hydatid cysts from other intracranial cystic lesions. For example, arachnoid cysts are typically extra‐axial and follow cerebrospinal fluid signal on all sequences, epidermoid cysts often demonstrate diffusion restriction, brain abscesses usually show rim enhancement and surrounding inflammatory changes, and cystic tumors often contain mural nodules or enhancing solid components. Recognition of these imaging clues is essential for accurate diagnosis and appropriate surgical planning [11]. Another important aspect highlighted by this case is the surgical principle of intact cyst removal. The Dowling technique was employed to facilitate gentle delivery of the cyst through controlled hydrostatic dissection while avoiding direct manipulation. Removal of the cyst without rupture is crucial because intraoperative rupture can lead to dissemination of protoscolices, recurrence, secondary cyst formation, or even anaphylactic reactions. The successful intact removal achieved in this patient demonstrates the effectiveness of meticulous surgical technique in preventing these complications [12]. Postoperative medical therapy and follow‐up surveillance are also key components of management. Albendazole therapy was initiated after surgery to eliminate potential residual parasitic elements and reduce the risk of recurrence. Regular clinical evaluation and follow‐up neuroimaging are recommended to detect recurrence or delayed complications at an early stage. This combined surgical and medical approach has been associated with favorable outcomes in pediatric intracranial hydatid disease [13]. Based on the literature, recurrence after surgical treatment of intracranial hydatid cysts is relatively rare when complete cyst removal is achieved without rupture; however, the risk increases in the setting of intraoperative spillage, incomplete excision, or secondary hydatidosis [14]. Compared to previously reported cases, this report is educational because the patient initially presented with nonspecific symptoms that were initially treated as migraine, highlighting the diagnostic challenge of intracranial hydatid cysts in their early stages. The case underscores the importance of maintaining clinical suspicion in endemic regions, recognizing characteristic imaging findings and applying meticulous surgical techniques to ensure intact cyst removal. These elements together illustrate key practical lessons for clinicians managing cystic brain lesions in children living in areas where echinococcosis remains endemic. Moreover, early diagnosis and timely surgical intervention have been shown to significantly improve neurological outcomes in pediatric patients with intracranial hydatid cysts [15].

## 4. Conclusion

In conclusion, this case highlights the diagnostic and therapeutic challenges of intracranial hydatid cysts, a rare manifestation of cystic echinococcosis, particularly in pediatric patients. A comprehensive approach integrating clinical evaluation, advanced neuroimaging, appropriate molecular confirmation, and meticulous surgical management is essential for optimal outcomes. Increased awareness among clinicians, radiologists, neurosurgeons, and parasitologists is crucial for early diagnosis and effective treatment. Furthermore, continued research into improved diagnostic tools and strengthened public health control programs is necessary to reduce the burden of hydatid disease in endemic regions.

## 5. Limitations

Histopathological confirmation of the excised cyst was not available in our case, and the diagnosis was primarily based on neuroimaging findings and clinical presentation. In addition, serological testing for echinococcosis was not performed. Furthermore, comprehensive systemic screening, including abdominal ultrasonography and chest imaging, was not undertaken to definitively exclude hepatic, pulmonary, or other organ involvement.

## Funding

No funding was received for this study.

## Ethics Statement

Ethical approval for this study was obtained from the Ethics Committee of Birjand University of Medical Sciences, Iran (Ethics Code: IR.BUMS.REC.1404.534). Written informed consent was obtained from the patient’s legal guardian (his parents) for the publication of this case report and any accompanying images.

## Consent

Please see the Ethics Statement.

## Conflicts of Interest

The authors declare no conflicts of interest.

## Data Availability

Data sharing is not applicable to this article as no datasets were generated or analyzed during the current study.
